# Practice status of specialized agencies for occupational health management of small- to medium-size enterprises and the factors improving their performance: a cross-sectional survey study

**DOI:** 10.1186/s40557-017-0161-4

**Published:** 2017-02-27

**Authors:** Saerom Lee, Jun-Pyo Myong, Eun-A Kim, Huisu Eom, Bowha Choi, Young Joong Kang

**Affiliations:** Korea Occupational Safety and Health Agency, Jongga-ro 400, Jung-gu, Ulsan, 44419 Republic of Korea

**Keywords:** Occupational health management, Specialized agency, Work performance, Workers’ health exam, Occupational health services

## Abstract

**Background:**

We examined the current status of specialized agencies for occupational health management (SAs) and their workforce. Furthermore, we aimed to clarify the current practice status of SA healthcare professionals and factors that influence their performance.

**Methods:**

To examine the current SA workforce, we analyzed data from the 2014 Survey of Current Status of SA and their Workforce from the Ministry of Employment and Labor (MOEL). Furthermore, we mailed out an original questionnaire to SA professionals to determine their current health management status and factors that affect their performance. Data from the respondents (*N* = 384) were analyzed.

**Results:**

In 2014, the workforce performing health management in SAs comprised 232 physicians, 507 nurses, and 312 occupational hygienists, with no significant regional differences in the distribution of physicians and nurses. According to the findings of the questionnaire, the average daily number of worker consultations by physicians and nurses was 22.8, while the average time taken for health management ranged from 74.3 to 104.3 min, depending on the size of the firm. Most of the respondents (41.5%) answered that they were following-up on more than 80% of individuals with illnesses. Among health management tasks, performance scores of “consultations for general diseases” and “consultations for lifestyle habits” were relatively high, whereas health promotion activities at workplaces were relatively low. There was a significant correlation between the utilization of general and special health examination results and task performance.

**Conclusion:**

Among health management tasks, follow-up management of individuals with illnesses and consultations for disease/lifestyle habits were relatively well performed, whereas health promotion activities at workplaces were not performed well. Among factors that positively influenced SA performance at workplaces, only the utilization of health examination results had significant effects. Therefore, to accomplish health management goals and perform effective health management at workplaces, there is a need to establish a comprehensive system of occupational health service outsourcing integrating health examinations and health management services. Furthermore, the current task system, which focuses on follow-up management, should be expanded to incorporate preventive and health promotion functions—the fundamental functions of occupational health services (OHS).

## Background

The occupational health service outsourcing (OHSO) system in the Republic of Korea was implemented as a measure to provide occupational health service (OHS) to workers of small- and medium-sized enterprises. This system allows owners of eligible businesses (determined according to the type and size of business as dictated by a presidential decree) to commission health management activities from certain institutions designated by the Minister of Employment and Labor [1]. This system was initiated after the success of the group health care test-implemented in the Masan Free-Export Zone in 1973, and the OHSO system was fully implemented after the amendment and promulgation of the Enforcement Decree of the Occupational Safety and Health Act (OSH Act) in July 1990 established legal grounds for specialized agency for occupational health services (SA) to take part in health management [2]. Through this Act, health management was expected to be performed more efficiently by commissioning the work from such a SA. Since 2014, there have been some amendments to the enforcement decree and enforcement regulations of the OSH Act, which changed some of the terms [3], but the overall scope of the health management tasks remained largely unchanged. According to the current Enforcement Decree of the OSH Act, all businesses (excluding those in the construction industry) that have less than 300 full-time employees or businesses that are located in remote areas designated by the Minister of Employment and Labor can outsource health management tasks to SAs.

The tasks of SA healthcare professionals are as follows: General disease consultation and care guidance- consultation and care guidance for general diseases, including hypertension, diabetes, dyslipidemia, liver disease, and obesity; occupational disease consultation and care guidance- consultation and care guidance for occupational diseases. Includes providing advices regarding abnormal findings in Workers’ Special Health Exam (WSHE), musculoskeletal diseases, and exposure to hazardous factors; lifestyle habit consultation and guidance- consultation and guidance regarding individual lifestyle habits. Includes providing advices regarding smoking, exercise, dietary habits, and drinking for the purpose of improving health of individual workers; health promotion activities in workplace I- includes tasks of surveying the health status within the workplace and establishing plans to promote health; health promotion activities in workplace II- includes operation of health promotion programs to assist smoking cessation/exercise/dietary habits/temperance/stress relief.

The OHSO system has been continuously expanded since its implementation, with 45 agencies being designated as SAs by the Ministry of Employment and Labor (MOEL) in 1994, 52 in 1995, 66 in 1998, and 101 in 2010; as of February 4, 2016, there are a total of 114 SAs carrying out health management in workplaces throughout the Republic of Korea. When calculated based on the designated cap for the management by one physician (i.e., 100 workplaces and 10,000 employees per one physician), then a total of 28,000 workplaces have outsourced their health management duties to SAs. Although the OHSO system has been credited with preventing industrial hazards and occupational diseases to a certain extent, there have also been some critiques of the system [4]. The most notable policy-related problems include the deregulation of the legal obligation for assigning occupational health physician, reduction in the mandatory number of health managers in workplaces, and abolition of the mandatory provisions regarding job training for health managers (Article 55 (2) of the Act on Special Measures for the Deregulation of Corporate Activities. Removed on August 3, 2007), all of which were brought upon by the amendment of the Act of Special Measures for the Deregulation of Corporate Activities in 1997 [4]. Problems related to the performance of health management remain as well. For instance, both SAs (i.e., the service providers) and workplaces alike have not been actively engaging in their health management tasks. Additionally, there are problems pertaining to the incongruence between the service recipients (employees) and service payers (employers), insufficient primary care due to prohibition of certain medical practices, and competition between SAs. In fact, a study surveying the perceptions of SA professionals has found that many are skeptical about the effectiveness of the OHSO system in managing health of employees [2]. Moreover, some people have raised concerns regarding the fact that most health management tasks are performed perfunctorily by SAs, which further undermines the effectiveness of the system [5].

Although there has been considerable research on the OHSO system until now, there has been no recent study on its current status. Furthermore, almost none of the past studies have endeavored to identify how these identified problems with the OHSO system actually influence the performance of health management. Hence, we surveyed the current status of SAs and their workforce, and identified their current practices and the factors that influence performance.

## Methods

### Subjects and materials

To survey the current status of SAs and their workforce throughout the Republic of Korea, we obtained and analyzed data from the 2014 SA and their Workforce Survey of the MOEL (data 1). The data included the information of SA’s address, number of workforce by job types, number of managing enterprises and employees, and so on.

Furthermore, in order to determine the current status of SA performance and the factors that influence performance, we performed a questionnaire survey of physicians and nurses working in SAs (data 2). As of 2014, there were 106 institutions designated as SAs. We contacted these agencies via phone calls and written notices before the initiation of the survey and mailed out questionnaires to all agencies. The questionnaire survey was conducted from June 26 to July 24, 2015.

Of the 106 SAs, we obtained respondents from 88 (83.0%); specifically, a total of 384 of the 807 questionnaires sent out were collected. Four of these collected questionnaires were excluded because of frequent missing responses, resulting in a total of 380 questionnaires for analysis. This meant that about 52.0% of the source population (739 persons) examined in the survey of SA designation by the MOEL in 2014 were included in the present study. More specifically, 28.0% of physicians in the source population and 62.1% of nurses participated in the questionnaire.

The items of the questionnaire were developed and selected by four healthcare professionals who worked for SAs as health managers via the Delphi technique. The items were designed to assess the current status of SA performance of health management and the factors affecting performance, and are broadly divided into items assessing agency status and those assessing individual performance. Only one representative from each agency was instructed to answer the items assessing agency status. Items assessing individual performance comprised the portion of specific health management tasks performed, follow-up care of individuals with illnesses and its effectiveness, performance scores of health management tasks, and utilization of health examination results. Performance scores of health management tasks were on a 10-point scale. Errors in the developed questionnaire were verified and corrected via a preliminary study.

### Statistical analysis

For the data 1 on the current status of SAs obtained from the MOEL and questionnaire survey (data 2), we calculated means, standard deviations, and percentages. For the data 2, linear regression analysis was performed to identify specific factors that affect health management performance. Model I is a Workers’ General Health Exam (WGHE) utilization and performance model adjusted for utilization of WGHE, total duration of career, number of firms visited per day, consultation numbers per day, time taken for health management at a workplace, and received education. Model II, WSHE utilization and performance model, was adjusted for utilization of WSHE instead of WGHE. All statistical analyses were performed using the SPSS Statistics 21.0 (IBM Corp., Armonk, NY).

## Results

### Current status of specialized agencies (SAs): analysis of data 1

On average, each SA managed about 80.9 and 78.5% of its designated caps for the number of firms and employees (data not shown). Regarding regions, SAs located in Busan managed the highest proportion of firms, on average, compared to the cap (98.0%), followed by those in Daegu (92.6%) and Ulsan (91.9%). SAs located in Jeonnam Province managed the lowest proportion of firms compared to its cap (72.9%). The proportion of actually managed employees out of the cap was highest in Seoul (91.7%), followed by Busan (89.5%) and Daegu (87.0%). The proportion of managed employees out of the cap was lowest in Gyeong-buk Province (66.9%; data not shown).

According to our survey of the SA workforce, the greatest numbers of physicians and nurses were working in Gyeonggi Province, wherein the greatest number of firms were managed. A total of 232 physicians performed health management at SAs in 2014, of which 108 (46.6%) were occupational and environmental medicine (OEM) physicians, 58 (25%) were preventive medicine physicians, and 68 (28.4%) were other types of physicians (i.e., physicians who are at least in their fourth year of residency in OEM or who have some experience in OEM). The number of firms and employees managed per physician was the highest in Busan Metropolitan City and the lowest in Gyeong-buk Province. The results were the same even when nurses were included in the calculation (Table 1).

**Table 1:** Status of workforce of specialized agencies for occupational health management by region (at the end of 2014)

	Number of agencies (%)	Physician				Nurse (%)	OH (%)	Managing enterprises/physician	Managing employees/physician	Managing enterprises/ HCP	Managing employees/HCP
		Total (%)	OEM	PM	Others						
Total	106 (100)	232 (100)	108	58	66	507 (100)	312 (100)	65.7	6319.5	21.4	2071.2
Seoul	8 (7.5)	25 (10.8)	15	3	7	47 (9.3)	29 (9.3)	56.0	6461.6	18.6	2197.3
Daejeon	4 (3.8)	8 (3.4)	6	1	1	14 (2.8)	9 (2.9)	60.2	6650.9	21.6	2379.6
Daegu	5 (4.7)	12 (5.2)	6	6	0	24 (4.7)	15 (4.8)	75.4	6977.3	25.7	2407.8
Gwangju	3 (2.8)	12 (5.2)	1	6	5	34 (6.7)	14 (4.5)	77.6	7131.4	20.5	1874.4
Incheon	10 (9.4)	18 (7.8)	11	2	5	34 (6.7)	25 (8.0)	60.2	5886.5	20.4	2001.1
Busan	3 (2.8)	7 (3.0)	4	1	2	14 (2.8)	8 (2.6)	84.8	7720.7	27.3	2468.3
Ulsan	4 (3.8)	22 (9.5)	8	4	10	59 (11.6)	31 (9.9)	73.0	6384.0	23.4	2039.4
Gangwon	3 (2.8)	4 (1.7)	3	0	1	6 (1.2)	6 (1.9)	62.0	5483.0	23.3	2050.6
Gyeonggi	29 (27.4)	56 (24.1)	16	18	22	120 (23.7)	80 (25.6)	66.9	6648.2	22.0	2192.8
Chungnam	8 (7.5)	14 (6.0)	6	1	7	28 (5.5)	18 (5.8)	72.6	6690.4	23.6	2190.8
Chungbuk	5 (4.7)	14 (6.0)	6	1	7	37 (7.3)	20 (6.4)	66.9	6018.0	21.5	1977.7
Gyeongnam	9 (8.5)	17 (7.3)	13	2	2	44 (8.7)	25 (8)	71.0	6682.1	20.4	1922.7
Gyeongbuk	11 (10.4)	18 (7.8)	11	7	0	36 (7.1)	24 (7.7)	53.0	4787.0	17.4	1582.3
Jeonnam	4 (3.8)	5 (2.2)	2	1	2	10 (2.0)	8 (2.6)	64.9	5600.3	21.6	1866.8

*OEM* physicians with board certification in occupational and environmental medicine, *PM* physicians with board certification in preventive medicine, *OH* occupational hygienist, *HCP* health care provider (including physicians and nurses)

In 2014, 187 of the 370 physicians (both physicians in charge of health management and physicians in charge of health exams in the same agency) working in SAs were in their 40s or younger (50.5%), followed by 73 in their 50s (19.7%), 31 in their 60s (8.4%), and 79 in their 70s or older (21.4%; data not shown). Gwangju had the highest distribution of physicians of age 70 or older (46.7%), followed by Gyeonggi Province (46.2%), Chung-buk Province (39.1%), Daegu (36.8%), and Ulsan (26.7%). In other regions, the proportion of physicians in their 70s or older was less than 10% (data not shown).

### Current practice status and factors affecting health management performance: analysis of data 2

We surveyed SA healthcare professionals’ current practice status and the factors influencing their health management performance. The general characteristics of the SAs and individual respondents are shown in Table 2.

**Table 2:** General characteristics of subjects

	Total N (%)	Physician n (%)	Nurse n (%)
Sex			
Male	54 (14.2)	50 (76.9)	4 (1.3)
Female	326 (85.8)	15 (23.1)	311 (98.7)
Age			
< 30	35 (9.2)	3 (4.6)	32 (10.2)
30–39	165 (43.4)	18 (27.7)	147 (46.7)
40–49	131 (34.5)	23 (35.4)	108 (34.3)
50–59	38 (10.0)	10 (15.4)	28 (8.9)
60–69	5 (1.3)	5 (7.7)	0 (0.0)
≥ 70	6 (1.6)	6 (9.2)	0 (0.0)
Total career length (yrs)			
< 1	37 (10)	6 (9.4)	31 (10.2)
1–4	152 (41.2)	24 (37.5)	128 (42)
5–9	73 (19.8)	9 (14.1)	64 (21)
10–14	38 (10.3)	9 (14.1)	29 (9.5)
15–19	39 (10.6)	11 (17.2)	28 (9.2)
≥ 20	30 (8.1)	5 (7.8)	25 (8.2)
Duration in current position (yrs)			
< 1	38 (10.0)	6 (9.2)	32 (10.2)
1–4	153 (40.4)	32 (49.2)	121 (38.5)
5–9	76 (20.1)	16 (24.6)	60 (19.1)
10–14	40 (10.6)	3 (4.6)	37 (11.8)
15–19	38 (10.0)	6 (9.2)	32 (10.2)
≥ 20	34 (9.0)	2 (3.1)	32 (10.2)
Type of employment			
Regular	317 (83.6)	42 (64.6)	275 (87.6)
Contract (full-time)	54 (14.2)	19 (29.2)	35 (11.1)
Contract (part-time)	7 (1.8)	4 (6.2)	3 (1)
Others	1 (0.3)	0 (0)	1 (0.3)

Table 3 shows the respondents’ current health management practice status. Physicians visited more firms in a given day on average (3.6 firms) than did nurses (2.6 firms). On the other hand, nurses, on average, consulted more workers in a given day. The average time taken for health management was 74.3 min for firms with less than 100 employees and 104.3 min for firms with more than 100 employees. In terms of the proportion of tasks performed, healthcare professionals engaged in employee consultation and management the most (42.7%), followed by administrative work within the agencies and preparation for firm visits (17.1%) and transit between agencies and firms (13.6%). Physicians were relatively more involved in employee consultation and management as well as other tasks (e.g., health exams, outpatient care, general ward care), while nurses were relatively more involved in administrative work within the SA in addition to employee consultation and management. For the item assessing the proportion of employees with illnesses who actually obtain medical care when instructed to seek it out, physicians most often answered that “a considerable proportion (about 60.0–79.0%) of the patients receive medical care” (38.5%), while nurses answered that “about half of the patients (about 40–59%) receive medical care” (31.2%). For the question asking whether SA professionals followed up on workers with illnesses when revisiting a firm, the majority (43.8 and 41.0%, respectively) of physicians and nurses answered that they followed up on most (about 80% or higher) of the workers with illnesses. In addition, 19.4% of the physicians and nurses indicated that they had not received education or training to enhance performance other than education for their licenses in the past year. A considerably higher number of nurses received Korea Occupational Safety and Health Agency (KOSHA) education while a relatively higher proportion of physicians received non-KOSHA education.

**Table 3:** Current status of health management practice

	Total	Physician	Nurse
Mean number of enterprises visited daily	2.7 ± 2.8	3.6 ± 4.8	2.6 ± 2.1
Mean number of workers consulted daily	22.8 ± 27.8	19.3 ± 13.2	23.5 ± 29.8
Mean time taken for health management (min)			
Firms with less than 100 employees	74.3 ± 22.7	74.3 ± 27.2	74.3 ± 21.7
Firms with more than 100 employees	104.3 ± 45.4	111.4 ± 87	102.8 ± 30.3
Mean daily transit time (min)	84.2 ± 147.3	69.3 ± 31.8	87.2 ± 161
Proportion of tasks performed (%)			
Employee consultations and management	42.7 ± 15.3	46.4 ± 20.1	41.8 ± 14.0
Administrative work within the agency, preparation for visiting workplaces	17.1 ± 9.9	10.3 ± 7.5	18.4 ± 9.8
Transit from agency to workplaces	13.6 ± 8.8	14.2 ± 10.4	13.4 ± 8.5
Taking minutes at workplaces and conducting consultations with owners	10.7 ± 6.6	7.1 ± 4.0	11.5 ± 6.7
Visiting workplaces	11.5 ± 8.7	11.2 ± 8.7	11.6 ± 8.7
Others	9.1 ± 10.7	17 ± 18.2	7.5 ± 7.3
Proportion of workers with illnesses who received hospital care (*n*, %)			
≥ 80%	45 (11.9)	10 (15.4)	35 (11.1)
60–79%	120 (31.7)	25 (38.5)	95 (30.3)
40–59%	111 (29.3)	13 (20.0)	98 (31.2)
20–39%	84 (22.2)	15 (23.1)	69 (22.0)
< 20%	14 (3.7)	0 (0.0)	14 (4.5)
Never	5 (1.3)	2 (3.1)	3 (1.0)
Proportion of workers with illnesses on whom follow-up was performed when visiting workplaces (*n*, %)			
≥ 80%	154 (41.5)	28 (43.8)	126 (41.0)
60–79%	129 (34.8)	23 (35.9)	106 (34.5)
40–59%	53 (14.3)	10 (15.6)	43 (14.0)
20–39%	26 (7.0)	1 (1.6)	25 (8.1)
< 20%	9 (2.4)	2 (3.1)	7 (2.3)
Received education other than that required for maintaining license within the past year (*n*, %)			
Never	71 (19.4)	12 (19.4)	59 (19.4)
KOSHA	197 (53.8)	14 (22.6)	183 (60.2)
Except KOSHA	185 (50.5)	47 (75.8)	138 (45.4)

*KOSHA* Korea Occupational Safety and Health Agency

There were no significant differences between physicians and nurses in the performances of any of the categories of health management tasks (significance not given). Performance scores of “general disease consultation and care guidance” (consultation and care guidance for general diseases, including hypertension, diabetes, dyslipidemia, liver disease, and obesity) and “lifestyle habit consultation and guidance” (consultation and guidance regarding individual lifestyle habits, including providing advice regarding smoking, exercise, dietary habits, and drinking for the purpose of improving health of individual workers) were high, with an over 8 points average for each, whereas that for “occupational disease consultation and care guidance” (consultation and care guidance for occupational diseases, including providing advice regarding abnormal findings for WSHE, musculoskeletal diseases, and exposure to hazardous factors) was relatively moderate, with an average score of 7.55. However, the performance scores for “survey of health status within firms and establish plans to health promotion” and “operate health promotion programs in firms (smoking/exercise/dietary habits/temperance/stress managing),” which both fall under the health promotion activities within firms, were relatively low (Table 4).

**Table 4:** Health management task performance

	General disease consultation	Occupational disease consultation	Lifestyle habit consultation	Health promotion in workplace (survey of status)	Health promotion in workplace (operation of health promotion programs)
Performance score (mean, 95% CI)					
Total	8.62 (8.49–8.76)	7.55 (7.37–7.73)	8.42 (8.26–8.58)	6.32 (6.09–6.54)	5.63 (5.33–5.92)
Physician	8.68 (8.37–8.98)	7.39 (6.84–7.93)	8.26 (7.81–8.71)	5.89 (5.24–6.54)	5.35 (4.60–6.11)
Nurse	8.61 (8.46–8.76)	7.58 (7.39–7.77)	8.45 (8.29–8.62)	6.41 (6.17–6.65)	5.68 (5.37–6.00)
Utilization of health exam results (mean, 95% CI)					
WGHE					
Total	8.85 (8.72–8.97)	7.38 (7.14–7.62) *	8.17 (7.98–8.35)	6.67 (6.43–6.91)	6.07 (5.77–6.38)
Physician	8.86 (8.60–9.13)	6.61 (5.84–7.39)	8.15 (7.72–8.59)	6.46 (5.78–7.15)	6.09 (5.37–6.82)
Nurse	8.84 (8.70–8.98)	7.54 (7.30–7.78)	8.17 (7.96–8.38)	6.72 (6.46–6.97)	6.07 (5.73–6.40)
WSHE					
Total	8.07 (7.85–8.29) **	8.00 (7.80–8.19)	7.08 (6.79–7.36) **	6.24 (5.97–6.51)	5.40 (5.08–5.72)
Physician	7.17 (6.40–7.94)	7.97 (7.48–8.46)	5.95 (5.10–6.81)	6.02 (5.32–6.71)	5.06 (4.26–5.87)
Nurse	8.26 (8.06–8.47)	8 .00 (7.79–8.22)	7.33 (7.04–7.61)	6.29 (6.00–6.58)	5.47 (5.12–5.82)

*WGHE* workers’ general health examination, *WSHE* workers’ special health examination

*p<0.05, **p<0.01

There were significant differences between physicians and nurses in the utilization of health exam results. GHE utilization were different in “occupational disease consultation and care guidance”, and SHE utilization were different in both of “general disease consultation and care guidance” and “lifestyle habit consultation and guidance” (Table 4).

According to the linear regression analysis, performance of all tasks significantly increased as the utilization of WGHE and WSHE results increased. In Model I, the performance of general disease consultation was positively related to having received education from KOSHA for a purpose other than license maintenance, but the results were not significant in Model II. Additionally, in Model I, the performance of occupational disease consultation was negatively related with a higher number of firms visited on a given day, but, as with general disease consultation performance, the results were not significant in Model II. In Model II, performance of lifestyle habit consultation was negatively related with a higher numbers of firms visited and employees consulted daily, even though the results were nonsignificant in Model I. In Model I, performance of surveying the firm for health promotion was significantly positively related to the number of employees consulted daily, although the results were not significant in Model II. Overall, only the utilization of WGHE and WSHE results had a significant effect performance of operating health promotion program (Table 5).

**Table 5:** Factors affecting performance of health management task (*ß*)

	General disease consultation	Occupational disease consultation	Lifestyle habit consultation	Health promotion in workplace (survey of status)	Health promotion in workplace (operation of health promotion programs)
Model I. WGHE and performance					
Total duration of career	0.040	0.033	−0.012	−0.020	0.012
Number of firms visited per day (≤5)	0.000	−0.095*	−0.055	−0.024	0.039
Consultation numbers per day (≤30)	0.066	−0.054	−0.046	0.084*	0.030
Time taken for health management at workplaces with less than 100 employees (>60 min)	0.001	−0.042	0.01	0.032	0.045
Time taken for health management at workplaces with 100 or more employees (>90 min)	0.056	0.013	−0.04	−0.038	−0.024
Received education at KOSHA (yes)	0.104*	−0.021	0.024	0.044	−0.008
Received education at other institutions (yes)	0.056	0.035	0.072	−0.042	−0.044
Utilized WGHE results	0.669***	0.582***	0.691***	0.671***	0.817***
Model II. WSHE and performance					
Total duration of career	0.067	0.013	0.021	−0.033	0.012
Number of firms visited per day (≤5)	0.007	−0.032	−0.104*	−0.016	0.033
Consultation numbers per day (≤30)	0.071	−0.083	−0.107*	0.080	−0.012
Time taken for health management at workplaces with less than 100 employees (>60 min)	−0.001	−0.023	0.006	0.062	0.041
Time taken for health management at workplaces with 100 or more employees (>90 min)	0.049	0.002	−0.064	−0.055	0.006
Received education at KOSHA (yes)	0.075	−0.058	−0.006	0.048	0.001
Received education at other institutions (yes)	0.066	0.008	0.081	−0.039	−0.034
Utilized WSHE results	0.381***	0.608***	0.415***	0.618***	0.702***

*WGHE* workers’ general health examination, *WSHE* workers’ special health examination

*p<0.05, ***p<0.001

## Discussion

We surveyed the current status of SAs and their health management performance. In doing so, we shed light on the SA tasks that are relatively well performed and those that are neglected, and determined those factors that are associated with performance.

In terms of the SA workforce in 2014, SAs in Gyeonggi Province appeared to have the greatest number of physicians and nurses. Notably, however, there were no significant regional differences in terms of per-physician and per-nurse numbers of firms and employees managed. Hence, the numbers indicate that there are few regional gaps in workforce supply (Table 1). The findings of the survey of physicians’ ages indicated that a considerable proportion of physicians who engage in health management are relatively older. Because we included all physicians working in an SA (i.e., both physicians who undertake health management and those who undertake health exams) for the age survey, the results may differ when the physician pool is limited only to physicians who perform health management. However, considering the amendments to the enforcement decree stipulating the qualifications for physicians who perform WSHE and those who perform health management in SAs [6], it is likely that the proportion of physicians aged 70 or older is much higher among the group of physicians who perform health management. In further support of this notion, when we exclusively analyzed 24 SAs that only perform health management tasks, 19 of the 36 physicians (52.8%) at these SAs were 70 years old or older (data not shown). Thus, it is likely that the proportion of physicians aged 70 or older among those who carry out health management tasks is higher than the 21.4% found in this study. Note that this is a very high number, particularly considering that only about 105 thousand out of the 5.56 million (about 1.9%) managers and professionals (physicians fall under this category as per the Korean Standard Classification of Occupations) in the Republic of Korea are 65 years old or older, according to the Korean economically active population survey [7].

In general, workplace health management comprises management of work and the working environment, health management, health consultations and health promotion, healthcare education, first aid training, healthcare information management, and risk assessment. Nurses’ duties include health management through health consultations and healthcare education; primary nursing services; overall operation of SAs and management and adjustment of them to promote occupation-specific business ties; health management for workplaces to maintain a disease- and hazardous-agent-free working environment; and record management. In contrast, physicians’ primary duties are assessment of health examination findings and protection of workers’ health through appropriate job allocations and transpositions and reducing working hours; survey of causes of health problems for workers and the implementation of medical measures to prevent recurrence; implementation of medical measures to maintain and improve workers’ health; health consultation, health education, and health improvement guidance for workers; inspection of workplaces and provision of guidance and suggestions; and inspection of causes of occupational diseases and ensuring the establishment of response measures. Despite delineation of these specific duties for both physicians and nurses, the health management tasks have not yet been standardized. In fact, there are various limitations (both temporal and spatial) preventing health managers from performing all of the tasks listed in the OSH Act. In our examination of the current performance of health management workers and proportion of tasks performed, we found that the time taken for health management was on average 74 min per one firm with less than 100 employees and 104 min per one firm with more than 100 employees. Furthermore, worker consultations and management accounted for the highest proportion of all tasks. However, health managers in general visited 2 to 3 firms on a given day and spent 30–50 min on average for worker consultation and management, depending on the size of the firm, while spending 1 h and 24 min for transit. Considering that they consult about 23 workers daily on average, it is assumed that health managers cannot select workers who require consultation and provide effective consultation in workplaces without workers’ health examination data (Table 3).

Regarding follow-up care, more than 43% of health professionals thought that over 60% of the workers with illnesses they had advised to seek medical care actually obtained it. Furthermore, more than 76% of health professionals responded that they had followed up on more than 60% of the workers with illnesses. Although the proportion of patients who seek medical care was merely a estimation by respondents, considering this finding in connection with the outcomes of follow-up care suggests that health managers are performing follow-up care to a certain extent (Table 3).

Health managers’ performance scores were, on average, higher than 5 out of a possible 10 points for all kinds of health management tasks. These scores are quite high, especially considering that the number of employees consulted daily was higher when a shorter time was taken for worker consultation per day. More specifically, tasks such as “general disease consultation” and “lifestyle habit consultation” were performed relatively well, and “occupational disease consultation” was also performed moderately well. However, further improvement is still needed for health promotion activities in workplaces. OHS fundamentally serves a preventive function [8]. In addition, the general goals of OHS suggest the principles of health promotion. Therefore, health promotion activities in workplaces should be a priority in OHS. These tasks—despite their importance—may be relatively underperformed in part because of the limitations of visiting management. In addition, as the commissioned health management tasks have not been standardized, the tasks that are relatively more impractical would be underperformed. Particularly, to carry out health promotion activities, company-wide environmental interventions would be crucial. Furthermore, team approaches, promotion of participation from the business owner and employees, and bidirectional communication play critical roles in these activities, which would further undermine performance of these activities in the current system of management on a short-term, visiting basis. Therefore, the scope of the commissioned tasks for health management should be clearly defined and specific tasks should be standardized, during which health promotion activities in workplaces should be acknowledged as a distinct category (Table 4).

The linear regression analysis showed that the numbers of firms visited daily and employees consulted daily were significantly associated with performance of health management tasks. However, it was difficult to conclude that these variables significantly affected performance, as the results were inconsistent across the models. On the other hand, utilization of health exam results consistently showed associations with performance of all tasks. Utilization of the WGHE results had a greater impact on performance of general disease consultation, lifestyle habit consultation, and health promotion in the workplace, whereas utilization of the WSHE results had a greater impact on the performance of occupational disease consultation. This was an expected finding in consideration of the target diseases for each task category and the nature of the examination items (Table 5).

When comparing the specific effects of utilization of health exam results on individual tasks, performance was higher among tasks that relied more heavily on utilization of health exam results, such as general disease consultation and lifestyle habit consultations (Table 5). Furthermore, many respondents noted in the comments section of the questionnaire that useful reference data for health management in firms is often scarce. This implies that, under the current OHSO system, health management is performed solely based on workers’ health exam results. Unfortunately, access is limited even to these data (i.e., the results of the WSHE and WGHE). As stipulated by the OSH Act, health examination institutions must send exam results along with a written follow-up management recommendation for employees with abnormal findings to employers. For the WSHE, reports for the statuses of all workers with abnormal findings within a firm and follow-up care recommendations are submitted, but even these only provide a brief note on the findings regarding a disease and follow-up care. The problem of limited accessibility is even worse for the WGHE results. Currently, workers are able to replace the WGHE with a health screening covered under the National Health Insurance (NHI) Act, which most workers end up doing. As such, health examination institutions that only perform the health screening covered under NHI and not the WSHE do not write up or submit follow-up management recommendations [9]. This would, in turn, further hinder health managers or occupational physicians from utilizing WGHE results. In other words, it appears to be difficult to access health examination results unless the health exam is performed at the same agency, and even when they are available, they do not necessarily provide detailed health-related information to workers. These problems appear to be more prominent in the service sector than in the manufacturing industry: whereas many workers in the manufacturing industry receive company-wide group health examinations, service sector employees individually receive examination at medical facilities close to their residences in place of a WGHE, thereby making it difficult for firms to receive follow-up care recommendations for group health management.

If health examination reports are the sole utilizable data under the current health management system, and if utilization of these reports is verified to increase performance, more efforts should be made to increase the use and accessibility of these data. Won et al. has found that the most highly demanded services by firms and employees were care guidance and management of individuals whose health examination reports had indicated an illness [10]. Therefore, in order to increase performance of such tasks and enhance accessibility of these data, a comprehensive and systematic OHSO must be developed by integrating health examinations and health management services. Furthermore, efficiency of health management in firms could be enhanced by providing services integrated with the measurement of the working environment and outpatient service for OEM.

However, only relying on health exam reports for health management of firms increases the risk of limiting the function of health management to follow-up care for employees. As such, the current OHSO system should be modified in order to ensure that the fundamental functions of OHS—beyond mere follow-up care of workers with illnesses—can be performed. In other words, OHS should take a step beyond care of individual workers with illnesses by instituting health promotion activities, which encompass activities that improve individual lifestyle habits and behaviors as well as those that improve working environments and organizational cultures via health education in workplaces. Previous studies have shown that reducing small risks among many people can prevent more disease than can reducing larger risks in a small number of high-risk people [11]. It seems appropriate for SAs to carry out health promotion activities in workplaces, but it would be difficult to realize the task under the current health management system, which is centered around the number of visits to firms. Regarding this matter, stakeholders should reach an agreement regarding changes to the current health management system or standardization of workplace health promotion projects.

This study also has some limitations. First, although we had distributed our questionnaire to all agencies, we only analyzed those that offered voluntary responses; in addition, there is a risk of non-respondent bias. Second, performance was measured based on a self-report questionnaire; as such, it was a subjective, rather than an objective, assessment. Third, as mentioned before, health management that relies solely on health exam results may limit the function of the management to follow-up care rather than preventive healthcare services. Thus, the performance findings must be interpreted carefully in consideration of the purpose of health management in workplaces. Finally, this study only examined physicians and nurses that work in SAs. In the future, studies should examine a wider pool of subjects to increase the validity of the results, as OHSO is performed not only by medical professionals (i.e., physicians and nurses) but also by occupational hygienists.

Notwithstanding these limitations, the present study is the first to survey and analyze the current status of health management performance by SAs and the factors influencing them. Particularly, this study shed light on the important fact that the current range of health management tasks is largely limited to follow-up care, instead of the much more important tasks of actively managing risk factors within the workplace. Therefore, it is critical to develop a more comprehensive OHSO system that could more effectively manage the overall processes of health management—namely, the prevention and diagnosis of diseases, and follow-up care. To this end, not only are efforts on the part of the SAs important, but also development of policies and support, such as standardization of health management tasks (including workplace health promotion activities and support for integration with other healthcare services), are required. Since its first implementation twenty years ago, the OHSO system in the Republic of Korea has maintained the same performance structure, and thus has continually been criticized for the same problems. Therefore, a standard guideline should be devised to improve the performance structure by reinforcing tasks that are performed well already and complementing tasks that are currently underperformed. Additionally, these notes should be reflected onto agency evaluations for a more practical and useful evaluation, which in turn would increase the quality of the OHSO system by preventing agencies from endeavoring to boost meaningless performance measures.

## Conclusion

This study sought to examine the current status of SAs and their workforce, and to identify the current practice status of SA professionals and the factors that appear to affect performance. Overall, there appears to be no or very few regional differences in the supply of SA healthcare professionals, with consideration of the number of managed firms and employees per healthcare professional. However, our results may suggest that there is a regional difference in age distribution for physicians, and that physicians who practiced health management tended to be in the older age groups.

Currently, the categories of tasks for workplace health management are as follows: health management, health consultation and improvement, health education, first aid instruction, and health information management. However, none of these tasks have been standardized yet. The findings of this study indicated that health managers are limited in their ability to perform these health management tasks when considering the number of employees consulted daily and the actual working hours. Whereas follow-up care for workers with illnesses is performed relatively well, workplace health promotion activities are relatively underperformed. Notably, utilization of health exam results was found to be associated with an increase in health management task performance, which was noteworthy because the other factors generally considered to influence performance were not found to be significant (e.g., number of firms visited daily, number of employees consulted daily).

In light of our findings, the current range of health management tasks is likely limited to individual interventions or follow-up care for workers with illnesses, and health exam results may be the only utilizable data. However, even these health exam results have low accessibility, which calls for efforts to increase such accessibility. One of the means of achieving these goals and effectively performing health management would be to establish a comprehensive and systematic OHSO system that integrates health management services with health examinations. Additionally, the current system, which is centered around follow-up care, should be modified to better perform preventive medicine and health promotion—the fundamental functions of OHS. However, this would be difficult to achieve, given the current system’s focus on the number of visits. Therefore, stakeholders should reach an agreement on matters regarding changes in the current OHSO system and standardization of tasks for workplace health improvement projects.

In conclusion, standardization of health management tasks, including those regarding workplace health promotion activities, and implementation of policies and support projects, such as integrative support with other healthcare services, must be realized to increase the quality of OHSO system and reinforce its effectiveness. In addition, these matters should be reflected in agency evaluations to increase the quality of the actual health management business.

## Abbreviations

KOSHA: Korea occupational safety and health agency
MOEL: Ministry of employment and labor
NHI: National health insurance
OHS: Occupational health service
OHSO: Occupational health service outsourcing
OSH Act: Occupational safety and health act
SA: Specialized agency for occupational health services
WGHE: Workers’ general health exam
WSHE: Workers’ special health exam
