# Erratum to: Practice status of specialized agencies for occupational health management of small- to medium-size enterprises and the factors improving their performance: a cross-sectional survey study

**DOI:** 10.1186/s40557-017-0175-y

**Published:** 2017-06-22

**Authors:** Saerom Lee, Jun-Pyo Myong, Eun-A Kim, Huisu Eom, Bowha Choi, Young Joong Kang

**Affiliations:** 1 Korea Occupational Safety and Health Agency, Jongga-ro 400, Jung-gu, Ulsan, 44419 Republic of Korea; 2 Department of Occupational and Environmental Medicine, Seoul St. Mary’s Hospital, College of Medicine, Catholic University of Korea, Seoul, Republic of Korea

## Erratum

After publication of the original article [1] the authors found that the following affiliations were incorrect at the time of publication:

1. Jun-Pyo Myong
  The current affiliation,’ Occupational Safety and Health Research Institute, Korea Occupational Safety and Health Agency’ is incorrect.
  The corrected affiliation should be:
**Department of Occupational and Environmental Medicine, Seoul St. Mary’s Hospital, College of Medicine, Catholic University of Korea, Seoul, Republic of Korea**
